# Health workforce development planning in the Sultanate of Oman: a case study

**DOI:** 10.1186/1478-4491-7-47

**Published:** 2009-06-11

**Authors:** Basu Ghosh

**Affiliations:** 1 Ministry of Health, Sultanate of Oman, Muscat, Oman

## Abstract

**Introduction:**

Oman's recent experience in health workforce development may be viewed against the backdrop of the situation just three or four decades ago, when it had just a few physicians and nurses (mostly expatriate). All workforce categories in Oman have grown substantially over the last two decades. Increased self-reliance was achieved despite substantial growth in workforce stocks. Stocks of physicians and nurses grew significantly during 1985–2007. This development was the outcome of well-considered national policies and plans. This case outlines how Oman is continuing to turn around its excessive dependence on expatriate workforce through strategic workforce development planning.

**Case description:**

The Sultanate's early development initiatives focused on building a strong health care infrastructure by importing workforce. However, the policy-makers stressed national workforce development for a sustainable future. Beginning with the formulation of a strategic health workforce development plan in 1991, the stage was set for adopting workforce planning as an essential strategy for sustainable health development and workforce self-reliance. Oman continued to develop its educational infrastructure, and began to produce as much workforce as possible, in order to meet health care demands and achieve workforce self-reliance.

Other policy initiatives with a beneficial impact on Oman's workforce development scenario were: regionalization of nursing institutes, active collaboration with universities and overseas specialty boards, qualitative improvement of the education system, development of a strong continuing professional development system, efforts to improve workforce management, planned change management and needs-based micro/macro-level studies. Strong political will and bold policy initiatives, dedicated workforce planning and educational endeavours have all contributed to help Oman to develop its health workforce stocks and gain self-reliance.

**Discussion and evaluation:**

Oman has successfully innovated workforce planning within a favorable policy environment. Its intensive and extensive workforce planning efforts, with the close involvement of policy-makers, educators and workforce managers, have ensured adequacy of suitable workforce in health institutions and its increased self-reliance in the health workforce.

**Conclusion:**

Oman's experience in workforce planning and development presents an illustration of a country benefiting from successful application of workforce planning concepts and tools. Instead of being complacent about its achievements so far, every country needs to improve or sustain its planning efforts in this way, in order to circumvent the current workforce deficiencies and to further increase self-reliance and improve workforce efficiency and effectiveness.

## Introduction

The Sultanate of Oman is a middle-income country on the southeast corner of the Arabian Peninsula, with a large shoreline from the Strait of Hormuz in the north to the borders of the Republic of Yemen [1]. It has a total land area of about 309.5 thousand square kilometres and a population of about 2.7 million in 2007, with about 30% expatriates. Countries such as Oman in the Gulf Cooperation Council (GCC) are net importers of their health workforce, but many of them have mounted national self-reliance initiatives prompted by (1) increasing competition for health workforce in the global market place, and (2) the urge to create more employment opportunities for citizens. According to some researchers: "The HRH issues in many Eastern Mediterranean Region (EMR) countries are not well-researched" [2].

The Sultanate of Oman's experience in health workforce planning and development may be considered an example of a remarkable initiative by a middle-income country in EMR. This paper takes stock of Oman's current status of workforce development vis-à-vis its past workforce problems, and narrates how the country is turning around its excessive dependence on imported workforce through systematic workforce planning.

## Case description

### Health workforce situation: past and present

The health workforce situation in Oman was unsatisfactory before the Omani renaissance in the early 1970s. The Sultanate had only 13 physicians and a few nurses in 1970. The physician-population ratio was abysmally low: two physicians per 100 000 people. Even in 1980, there were only 514 physicians and 1096 nurses. At that time, there were only 5.1 physicians per 10 000 people. There were hardly any Omani health professionals in 1970, and only a few in 1980.

**Figure 1:**
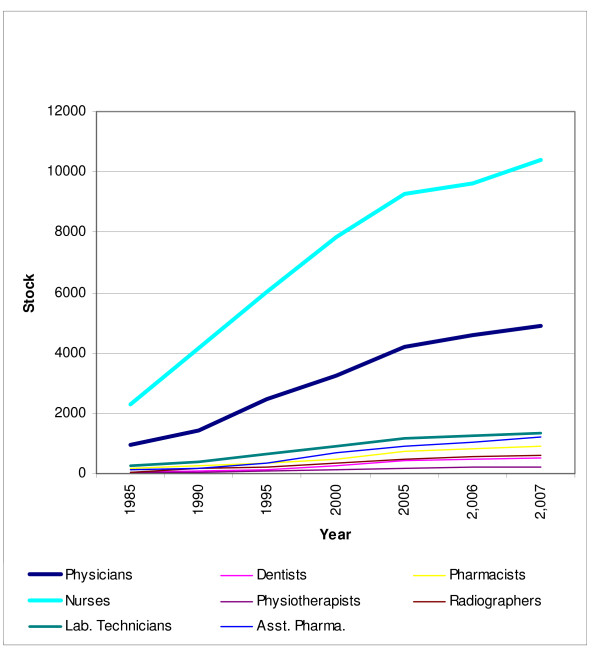
Growth of health workforce stock in Oman, 1985–2007

The physician, nurse and most other professional categories in Oman have grown substantially during 1985–2007, as may be seen from Table 1. Figure 1 depicts the significant rise in the numbers of physicians and nurses in Oman during this period. This growth was necessitated by expansion or upgrading of the health care infrastructure. The Sultanate undertook that task through systematically formulated five-year health development plans.

**Table 1:** Health workforce stock in the Sultanate, 1985–2007

**Category**	**Year**						
	1985	1990	1995	2000	2005	2006	2007

Physicians	958	1441	2477	3258	4182	4579	4908

Dentists	53	84	143	262	448	496	524

Pharmacists	193	247	356	496	753	805	916

Nurses	2288	4147	6036	7829	9277	9615	10 394

Physiotherapists	44	50	69	150	161	198	232

Radiographers	64	161	232	334	480	550	593

Lab. Technicians	247	408	670	910	1169	1258	1331

Asst. pharmacists	112	186	367	688	912	1028	1200

Source: Annual Health Information Report 2007, Ministry of Health

**Figure 2:**
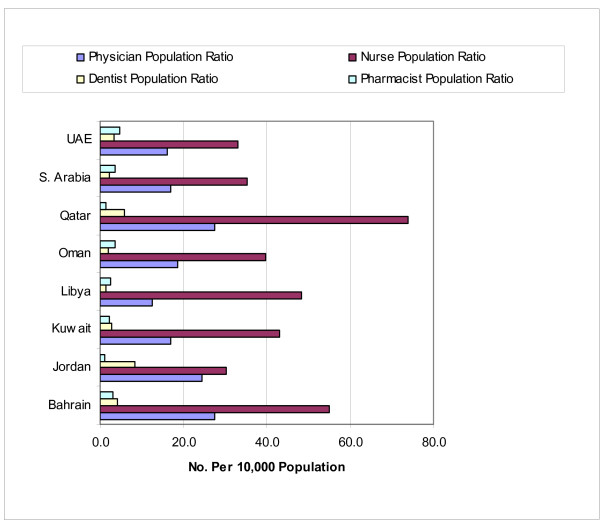
Workforce density comparisons.

Substantial growth in health workforce stocks enabled the Sultanate to step up its workforce population ratios to reach satisfactory levels fairly comparable to those of other countries in the region, as may be seen in Figure 2, which presents intercountry comparisons [3]. The physician-population ratio grew from 11.8 per 10 000 people in 1985 to 17.9 in 2007. The nurse-population ratio grew in the same period from 28.9 to 37.9. Since Oman is still a net importer of health workforce, the Sultanate's achievement in building up its health workforce stocks can be fully appreciated only if one considers the growth in health workforce along with its increased self-reliance in workforce.

The total stock of physicians employed by the Ministry of Health (MoH) grew 5.4-fold during 1985–2007 (from 638 to 3459). During the same period, the number of nurses grew 4.5-fold (from 1947 to 8143). As is evident from Table 2, the stocks of most other professional categories also grew during 1985–2007.

**Table 2:** Health workforce stocks in Ministry of Health, 1985–2007

**Category**	**Year**							Growth*
		
	1985	1990	1995	2000	2005	2006	2007	
Physicians	638	994	1800	2253	2981	3252	3459	5.4

Dentists	23	40	77	106	168	179	190	8.3

Pharmacists	22	33	63	78	154	178	196	8.9

Nurses	1947	3512	5128	6619	7909	8143	8680	4.5

Physiotherapists	24	32	56	120	123	145	151	6.3

Radiographers	76	123	183	268	401	458	488	6.4

Lab. technicians	206	323	513	707	873	936	1000	4.9

Asst. pharmacists	114	160	345	479	690	776	840	7.4

*MoH stock in 2007 relative to stock in 1985 (times).

Source: Annual Health Information Report 2007, Ministry of Health

However, these gains in workforce stocks were not achieved at the cost of loss in workforce self-reliance, as is apparent from Table 3. On the contrary, self-reliance, as measured by the percentage of Omani in the workforce, grew substantially during the period. The overall Omanization level in the MoH grew from about 52% in 1990 to 68% in 2007. Even in the case of leading categories such as physicians, nurses, laboratory technicians, etc., the Omanization level increased steadily over the plan periods.

**Table 3:** Progress in Omanization in MoH during 1990–2007

**Category**	**Percentage Omani by end of year**				
	
	1990	1995	2000	2005	2007
Physicians	8.7%	12.8%	18.7%	27.3%	29.0%

Dentists	25.0%	16.9%	30.2%	41.1%	42.0%

Pharmacists	21.2%	12.7%	28.2%	48.7%	54.0%

Nurses	11.6%	14.5%	35.9%	59.2%	64.0%

Physiotherapists	18.8%	62.5%	71.7%	64.2%	68.0%

Radiographers	13.8%	28.4%	47.0%	59.6%	60.0%

Lab. Technicians	19.5%	31.0%	36.9%	51.7%	55.0%

Asst. Pharmacists	9.4%	26.1%	50.5%	69.3%	67.0%

Overall	51.9%	50.1%	53.5%	65.8%	68.0%

Source: Annual Health Information Report 2007, Ministry of Health

However, in certain categories Omanization witnessed a slight fall in 1995 over 1990, as this period saw the establishment of major regional hospitals. With regard to teachers/tutors, this period marked the establishment of regional nursing schools and several allied health professional courses, which resulted in the importing of specialized teachers in the respective fields. This explains why the Omanization ratio of teachers fell during 1991–1995. In fact, the Sultanate as a whole has emerged from the stage where it used to rely heavily on workforce imports to be able to extend its health care infrastructure.

As may be seen from Table 4 relating to the whole country, at present 58% of the Sultanate's health workforce is Omani. About 25% of its physicians and 55% of its nurses are Omani. However, the overall Omanization level (i.e. percentage of Omani) is higher (68%) in the MoH, the principal health care provider. The current Omanization levels regarding physicians and nurses in the Ministry of Health are 29% and 64%, respectively.

**Table 4:** Omanization status in health subsectors by category, 2007

**Category**	**MoH**	Other Govt.*	**Private sector**	**Oman**
Physicians	29.0%	53.9%	2.3%	24.6%

Dentists	42.0%	84.2%	1.0%	18.9%

Pharmacists	54.0%	65.8%	0.1%	14.3%

Nurses	64.0%	19.9%	6.5%	55.4%

Physiotherapists	68.0%	90.0%	3.9%	56.5%

Radiographers	60.0%	56.0%	1.8%	54.3%

Lab. technicians	55.0%	66.7%	0.9%	46.5%

Asst. pharmacists	67.0%	54.8%	6.6%	50.8%

Overall	68.0%	54.6%	4.8%	58.1%

*Includes SQU Hospital, Petroleum Development Oman and Royal Oman Police, excludes Armed Forces.

Source: Annual Health Information Report 2007, Ministry of Health

It is observed that the Omanization level in the MoH is lower for the key category of physicians, while it is higher for nurses and other categories. This can be explained by the fact that the MoH itself produced nurses and other paramedical categories and gave priority to its own employment of such personnel, while also accommodating the demands of other public sector entities for such personnel as much as feasible. That is why the private sector has achieved a low level of Omanization in the nursing and paramedical categories.

**Figure 3:**
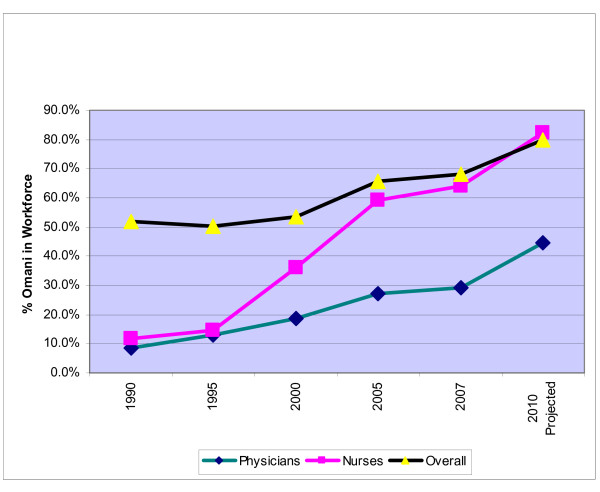
**Self-reliance in physicians and nurses, 1990–2007**.

However, the MoH had to depend on the Sultan Qaboos University (SQU) to produce physicians, who were demanded by the entire health sector. The private sector got only a very low share of Omani physicians, since they preferred to work in the public sector due to certain perceived advantages. However, senior Omani physicians employed in the public sector do work as part-time consultants to private health establishments during their off-duty hours, with the approval of the Government. This explains why the private sector has achieved a low level of Omanization in the physician category. The trends in the growth of workforce self-reliance can be seen in Figure 3.

### Development of health professional education

It was only in 1959 that health professional education saw a modest beginning in Oman. The Ministry's first major health sciences educational institution was established in 1982, initially to run a medical laboratory sciences course. Subsequently, it started courses in radiology, physiotherapy and dental surgery assistance. Health professional education got a major boost with the preparation of Oman's strategic health workforce development plan in 1991. Regional nursing institutes were set up in 1991 all over the country so as to ensure equitable opportunities for admission to all students across the Sultanate. This regionalization policy was designed also to ensure proper distribution of nurses in different health regions.

The Ministry also set up new institutes for education in other allied professions such as public health, pharmacy assistance, medical record technology, etc. When basic education reached a reasonably satisfactory status, the MoH placed emphasis on postbasic education in selected nursing specialties. It set up an Institute of Specialized Nursing in Muscat to serve as the focal centre for nursing specialty education in the Sultanate. It also initiated specialized training in midwifery in Muscat and a few regional capitals. By the end of 2007, the MoH had built a stock of 3164 nurses working in specialized areas, yielding 56% Omanization among specialized nurses.

SQU has made considerable headway with its Bachelor of Science in Nursing (BSN) programme, and the Nizwa University has already initiated its BSN course. The Ministry of Health, apart from sending its staff (diploma holders/graduates) to acquire BSN/MSN degrees from reputed universities abroad, has also developed collaborative arrangements with overseas universities for locally producing BSN graduates in some of its own institutes.

As may be seen from Table 5, the country's educational infrastructure grew substantially within only a few years. As of 2007, more than 2100 students were studying general nursing (more than eight times the number in 1990). Further increase in the intake of MoH nursing schools is neither required (as the Ministry has already achieved a high level of Omanization) nor desirable (as clinical practice for more nursing students is a constraint now, since the universities have also started nursing degree courses). As many as 630 students earned their basic diplomas in a health profession from these institutes during 2007 (about 15 times the number in 1990). In all, about 8400 students have graduated from MoH institutes over the years. General nursing graduates represented about 72% of all graduates.

**Table 5:** Health professional educational institutes, 2007

**Type of Institution**	**Number**	**Total intake**
**Degree-awarding institutions**		

College of Medicine	2	203
College of Dentistry	1	60
College of Pharmacy	1	58
College of Nursing	1	50
College of Lab. Technology	1	40
**Diploma-awarding institutions**		
Nursing schools	11	545
Midwifery schools (Postbasic)	3	62
Paramedical training institutes	4	208
School of specialized nursing (postbasic)	1	106

Source: Ministry of Health, Oman

SQU, the Sultanate's first university, began medical education in Oman in 1986 with 45 students. In all, 1053 students earned their MDs from SQU during 1993–2007. A private medical college, Oman Medical College (OMC), was established in 2001 with an intake of 69 students. This college is permitted to use some of the Ministry's regional hospitals for clinical instruction and practice. The MoH actively collaborates with and supports SQU in numerous ways. SQU makes use of many of the Ministry's major hospitals for clinical practice and internship.

The authorities considered proposals for setting up a dental college in the public sector and another in the private sector. But, after detailed considerations supported by a policy brief prepared by the Health Workforce Planning Team, only one private dental college (Oman Dental College) was approved. This college is permitted to use some of the Ministry's hospitals as its teaching hospitals. Several other private-sector initiatives in health workforce production have also taken place (such as courses for medical secretaries and pharmacy assistants).

Postgraduate medical education commenced in Oman with the establishment in 1994 of the Oman Medical Specialty Board (OMSB) as the highest supervisory body of postgraduate medical training programmes in Oman. The Board developed postgraduate residency programmes in the country with the active support of the Ministry of Health, SQU and other constituents. The MoH, SQU and other employers sponsor Omani candidates in various specialties under OMSB or for overseas education/training. Many residents have already cleared all requirements of the OMSB and international boards/colleges, and earned their full membership in such bodies or earned their master's or doctorate degrees. The Omani stock of medical specialists rose to 225 at the end of 2004 and is projected to rise to 459 at the end of 2010. Overall self-reliance in the medical specialists subcategory is expected to rise from 22% at the end of 2004 to 32% at the end of 2010 [4].

**Figure 4:**
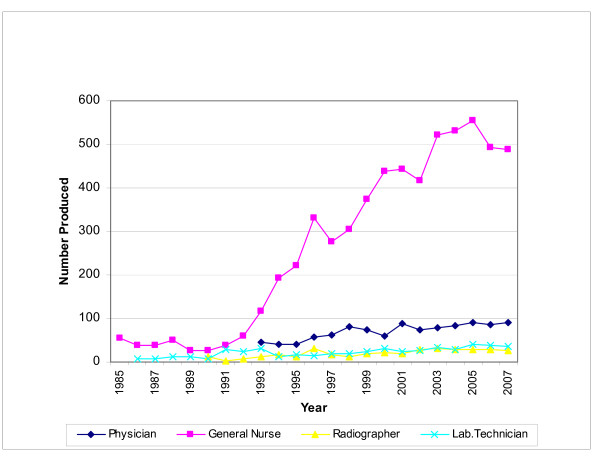
**Health workforce production in select categories, 1984–2007**.

The trends in the growth of health workforce production achieved through the building up of Oman's health professional education infrastructure are evident from Figure 4. In addition to quantitative growth in workforce production, the Ministry of Health has also focused on qualitative improvement of the outputs of the educational system. Curricula of educational programmes run under its auspices were reviewed and improved periodically with the support of teachers, service institutions and international consultants.

### Continuing professional development

The Ministry has initiated steps for strengthening the organization of continuing professional education (CPE). Staff development and CPE functions at the autonomous hospital level have been re-engineered in tune with the guidelines on hospital autonomy. To provide leadership in further developing this area, the Ministry has established a central steering committee for CPE at the national level. This committee has spearheaded the development of a sound CPE policy and an accreditation system for CPE. The Ministry has set up a countrywide CPE infrastructure. Staff development units have been established in all autonomous hospitals. Regional CPE Committees have been formed in all MoH regional directorates. The Ministry's Directorate of Continuing Education has been revitalized, with the responsibility to coordinate CPE at the national level.

### Workforce management systems

The Ministry of Health has persistently attempted to fine-tune workforce management within the constraints set by the civil service law, other laws and regulations of the government. It has set up computer-based information management system at all levels, covering all aspects of health system management – including workforce management – so as to ensure better availability and reliability of information for more rational decision-making on workforce management. Corrective actions have been taken to streamline the recruitment system and minimize the recruitment delay. The MoH has issued a series of guidelines on hospital autonomy and introduced organizational and managerial reforms leading to a re-engineering of hospital management in Oman [5,6]. These guidelines have paved the way for effective decentralization of health administration and workforce management at the hospital level.

### Planning process and development

The government of the Sultanate of Oman has viewed human resources development in all sectors, including the health sector, as an integral part of the Omani economic and social development. It has advocated a national self-reliance or Omanization policy as a political necessity and as the main underlying force in workforce development in all sectors. In view of this, the Ministry of Health has attached strategic importance to health workforce development as essential for achieving self-reliance in the workforce.

As seen from the preceding discussion, in the early 1970s, when the Omani renaissance began, the Sultanate did not have enough educated workforce to mount economic development initiatives. The country's health development plans emphasized speedy development of the health care infrastructure, health services and health programmes. In order to achieve this goal, it began to import a health workforce. Such a policy stance was necessary at that time, so that Oman could improve the health status of its people even before the country had its own critical mass of health workforce. However, for sustainable health development, it was considered politically expedient to stress the simultaneous development of its own national health workforce. This initiative also held potentials for employment generation for the rapidly growing educated Omani population consequent to massive growth in education resulting from substantial investment to create an educational infrastructure.

### Strategic planning

While formulating the fourth health development plan (1991–1995), the Ministry of Health felt the need to attach specific importance to health workforce planning. The MoH invited a WHO consultant (based in a school of business and with proven international expertise in health workforce planning) to help prepare a strategic health workforce development plan. This initiative resulted in the preparation of a long-term perspective plan for workforce development as a supplement to the Fourth Five Year Health Development Plan 1991–1995 [7].

This broad programming for workforce development, undertaken in 1991, set the stage for adopting health workforce planning as an essential strategy for sustaining health development and achieving self-reliance in the health workforce. The report presented the first-ever comprehensive health workforce situation analysis for the Sultanate, formulated a long-term production plan under alternative scenarios, and came up with significant recommendations that eventually paved Oman's march towards health workforce development and self-reliance. MoH accepted the recommendations and decided to attach strategic importance to workforce planning as the basis of health workforce development in Oman.

### Workforce development strategy

Since the Sultanate adopted Omanization as a national strategy governing all sectors of the economy, including the health sector, health workforce development had to be undertaken in tune with health sector development. As the country developed its health care infrastructure, it needed a bigger workforce. There was also a political compulsion to increase the Omanization level among the health workforce. The only way to strike a balance between these two counteracting forces was to adopt a sound approach to health workforce planning. This is what the Sultanate decided to do.

Such a strategy could succeed only if the country produced a big enough workforce at least to meet the new demand for workforce (necessitated by the expansion or upgrading of the health care infrastructure). It was also necessary to ensure that a health care institution has just the quantity and quality of workforce that it needs, neither more nor less. The health workforce assigned to a health institution should be enabled to give its best to the organization, and for that there was a need for a sound workforce policy and adequate organizational support, including equipment and other material support, building, transportation, etc. In this context, MoH adopted the following workforce development strategy:

• Form an interdisciplinary team for workforce planning.

• Develop effective workforce policies.

• Undertake careful category-wise production planning.

• Develop needed educational infrastructure in the national capital and in regions.

• Produce the required workforce of satisfactory quality.

• Collaborate with universities/specialty boards/councils in Oman and abroad.

• Ensure continuing professional development of staff.

• Improve workforce management systems.

### The workforce planning team

A core team, led by the Ministry's health workforce planning advisor, was constituted with the staff of the directorate of planning to help formulate workforce policies/programmes and related health care policies, and prepare strategic and detailed workforce development plans [8]. The team comprised a workforce planner (a health management professor with statistics and social science background and specialized in health workforce planning), a human resources management (HRM) professional (with a master's degree in the field), a physician (with a master's degree in health management and with an interest in HRM) and a qualified industrial engineer (with an interest in workforce planning and related productivity issues).

The team leader was given direct access to the Minister of Health and other policy-makers, who took keen interest in workforce development planning. In order to ensure broad professional involvement of all concerned, the planning team felt it necessary to maintain close linkage with other top- and mid-level management personnel in health affairs, planning affairs, education and training affairs and administrative affairs. The team leader made it a point to interact closely with all relevant officials of the Ministry, such as national and regional directors general; key directors, such as directors of nursing affairs and personnel; hospital executive directors; medical department and nursing heads; and deans of medical schools, nursing and allied health institutes, in the context of specific plans and programmes.

### Workforce policy development

The Ministry of Health is required to conform to the civil service law and other regulations laid down by the Ministry of Civil Service. However, it is free to develop its own perspectives and approaches in order to optimize the human resources system, without violating the laws of the land and various government regulations. The MoH issued its first official guidelines on health workforce development, which stressed that health workforce planning was vital to the development of the Omani health care system. It stated that the planning approach would be based on the following principles: (1) the need to develop a critical mass of Omani personnel in all key professional categories, (2) the need to ensure adequate and appropriate workforce availability in various categories and in different health institutions/regions, and (3) the need to achieve high workforce productivity through optimum utilization of the workforce [9].

### Workforce production planning

The MoH prepares five-year workforce development plans as part of the health development plan. These plans are undertaken according to the following steps:

• Assess the macro situation and delineate the strategies for workforce development during the five-year plan.

• Periodically undertake category-wise detailed perspective planning for all major categories, e.g. physicians (including specialists and subspecialists), nurses (including specialist nurses), radiographers, assistant pharmacists, physiotherapists, sanitarians, laboratory technicians and pharmacists.

• Advise the Ministry on further steps for education and training of physicians, nurses and other professionals and for planned Omanization of these key categories.

Detailed studies were undertaken for perspective planning of the physician and nursing categories, including medical specialties/subspecialties and nursing specialties, as well as other allied professions. These studies projected estimates of future requirements under alternative scenarios, and helped the Ministry to decide on its strategies and plans for further workforce production in the country for gradual and smooth Omanization of these categories. Studies were also undertaken for estimation of fellowship requirements for overseas education, as a tool for mobilizing more fellowship resources. Oman's approach to workforce development planning has already been cited in the literature [10].

### Use of planning tools

Workforce planning techniques and tools are abundantly available today, thanks to the work of WHO and other pioneers in the area [11-14]. However, there is always a need to adapt such tools and techniques to a country's specific requirements and policy emphasis. In keeping with this felt need, the Ministry's planning team developed its own tools for category-wise workforce planning, and hospital/primary health care workforce requirement planning. It has also employed other approaches to workforce planning such as the use of the Delphi technique for subspecialty priority planning, in-depth interviewing to assess development potentials of staff, holding multi-level workshops for consensus-building for hospital autonomy policy development, etc.

The MoH has developed its category-wise workforce planning model with a focus on self-reliance [15]. The model was later modified to make it a user-friendly computer model for the Ministry's internal use. The Ministry has repeatedly used this model for long-range workforce planning for category-wise production decisions, apart from using MS Excel-based tools for macro-planning of categories in the context of five-year workforce planning.

Initially, hospital staffing decision-making in the MoH was based mostly on the demands of hospital administrators and heads of departments, which in turn emanated from perceived allocation needs and no systematic considerations of workload. This approach was subsequently modified in tune with the WHO's workload indicators of staffing needs (WISN) approach [16]. However, this technique was soon found to be deficient for application to specialized physicians, nurses and other staff categories in multispecialty hospitals. The applicability of WISN in nursing has also been questioned by the nursing profession [17]. Although not all of those deficiencies could be wished away, the Ministry developed its own approach to hospital workforce requirement planning based on productivity-cum-allocation considerations [18]. The methodology adopted by the Ministry of Health consists of the following steps:

• Whenever a new project is initiated (or a serious demand for additional resources is made by an existing unit), and there is a prima-facie case in the opinion of the Minister of Health, the workforce planning team undertakes an objective workforce requirement planning study.

• The planning report is submitted to the policy-makers for their consideration and approval.

• After approval of the report by the policy-makers, financial approval is solicited from the Ministry of Finance and Economy.

• After the necessary workforce is approved and finance allocated, the workforce planning report is used as an important reference document for the hospital.

• If feasible, the study is repeated once every three years, or earlier if the hospital administration perceives major workforce inadequacies.

In view of the need to use such models repeatedly to monitor and improve the workforce situation in institutions, the Ministry further modified the hospital workforce requirement planning approach to build user-friendly, computer-based models [19,20].

The planning team continued to apply this tool for staffing of new or upgraded regional hospitals, as well as for reassessing the workforce situation in existing hospitals. Subsequently, this approach was extended to undertake workforce planning for primary health care institutions as well [21]. Flexible rational staffing patterns (norms) were developed for local hospitals and health centres, and the Ministry circulated these to all concerned, as the guidelines for staffing primary health care institutions. This decision paved the way for the Ministry's concerted efforts to improve the delivery of primary health care.

### Issues successfully resolved

The lack of local expertise in workforce planning initially stood in the way of the realization of the desire of policy-makers to systematically plan health workforce development. The MoH persisted in trying to recruit a consultant in this field through WHO. Several consultants were assigned to the Ministry, but none seemed to have understood the vision of the policy-makers. WHO finally succeeded in arranging a suitable consultant in this field and his report was accepted by the Ministry as the blueprint for further action in this area.

The proliferation of categories and job titles made workforce planning extremely difficult in the early days of workforce planning. As a first step in the strategic workforce planning exercise, the WHO short-term consultant (STC) undertook, in collaboration with a very high-level team, a categorization of health workforce in order to organize health workforce information as an aid to planning [7]. This enabled proper data generation and compilation as part of the Ministry's health information system.

Countries dependent on imported manpower need a planning model suitable for their own settings. As stated before, this problem was resolved by the WHO-STC through developing a simple quantitative model focused on self-reliance, or Omanization [7,15]. This model also enabled study of the possible impact of various production plans on Omanization under alternative scenarios.

Having realized the crucial importance of workforce planning after the strategic planning exercise, the MoH felt the need for a long-term consultant in workforce planning to help develop its health workforce development initiatives. It approached WHO to appoint the WHO-STC for a long-term assignment to Oman. As WHO was unable to accede to the request of the Ministry, the policy-makers persuaded the STC to accept a direct appointment as an advisor with the Ministry. After initial difficulties, the STC finally joined MoH to serve on a long-term basis. Thus, the Ministry ensured continuous availability of health workforce planning expertise to undertake planning as well as counterpart training in this area.

The Ministry had to make a careful decision as to where to locate the advisor in the organizational hierarchy. The Ministry's human resources function was dispersed mainly under four directorates-general: the Directorate-General of Planning (workforce planning), the Directorate-General of Education & Training (workforce production and training), the Directorate-General of Administration (recruitment and employment etc) and the Directorate-General of Health Affairs and the regional directorates-general (workforce utilization).

As an organizational imperative, the Ministry placed the health workforce planning advisor in the Directorate-General of Planning. The advisor's direct contact with the highest policy-making level enabled the planning team to translate the policy-makers' vision in terms of concrete workforce development plans with their active encouragement and engagement. The policy-makers began to refer all important health workforce development and related issues to the advisor for special studies, analyses and advice. The workforce planning team started functioning like a management services department in a corporate entity.

Yet another problem faced by the Ministry was how to improve the quality of staff it employs. In the initial phase of Oman's renaissance, the Government, in its urge to create employment opportunities, undertook extensive recruitment, although educational development up till then was inadequate. Because of this, the MoH had to recruit many staff with insufficient educational preparation.

A couple of decades later, the Ministry faced a skill-mix mismatch problem. In response to this issue, and at the instance of the policy-makers, the workforce planning team took up several time-consuming studies on assessing development potentials of Omani and non-Omani employees of important HQ Directorates-General through in-depth, semistructured personal interviews and analysis of personnel files. As a spin-off of these studies, the Ministry took specific actions to streamline some key components of workforce policies. It also took important individual-level actions to develop further potential of Omani employees.

Another issue usually faced by the Ministry is how to ensure that a hospital gets exactly the number of staff that it requires, rather than what it demands. When the MoH started upgrading the regional hospitals, it started receiving many requests for additional staff. In the past, the Ministry assigned staff to institutions on an ad-hoc basis, following demands of heads of departments. In response to this dilemma, the workforce planning team developed a unique approach, as mentioned earlier. The adoption of this rational staffing policy has enabled the MoH to optimize expenditure on the health workforce, on which health care systems around the world spend 60% to 80% of their recurrent budgets. Oman's experience in staffing policy development was described by WHO analysts as suggestive of how "having policy-makers involved at the start helps to ensure use of data for policy development and implementation" [22].

The Ministry wanted to ensure that awareness and appreciation of the importance of workforce planning were high among its senior management personnel. When workforce planning was first initiated, many of the top- and mid-level functionaries were not so familiar with the concept of health workforce planning. On the policy-makers' special initiative and with their active support, the newly inducted advisor organized a top-level workshop for the Ministry in 1992 in the form of a retreat in scenic surroundings in the south, miles away from the national capital.

The objective of this workshop was to expose the national and regional directors-general to the concepts and tools of health workforce planning. This interactive two-week workshop, organized along WHO guidelines, helped to generate awareness of and interest in health workforce planning among the top echelon of the Ministry's health administrators [12,23].

In the years following this workshop, the workforce planning team engaged in a series of planning exercises and special studies identified by the policy-makers. These studies further helped to increase the knowledge and appreciation of workforce planning.

The policy-makers felt the need to orient the high and middle-level administrators to workforce policies and policy-making processes. To address this concern, the planning team conducted a highly interactive first national workshop on workforce policy in 2001 [24]. The workshop, held under the joint auspices of the MoH and WHO, presented a unique opportunity for broad-based consultation among health services administrators, educators and workforce managers for further development of the Ministry's workforce policies, programmes and systems. The Minister of Health encouraged the participants by attending the inauguration and the closing sessions and listening to the recommendations of the participants. The workshop finally led to the formulation and release of the first official guidelines on health workforce development at the Ministry of Health [9].

The policy-makers desired to assess the adequacy of ad-hoc training programmes. As desired, the workforce planning team studied the effectiveness of the Ministry's training system at the hospital level. This brief study revealed the intrinsic inadequacies of such ad-hoc training practices, confirming widely-held perceptions that such training of personnel was not effective. These findings, coupled with the observations of some visiting consultants, prompted the MoH to mount a strong CPE system [25,26].

It was also necessary to examine the adequacy of the workforce management information system. Towards that end, a study was undertaken to evaluate and ensure availability of reliable information for rational decision-making on workforce management [27]. An interdisciplinary team took stock of all the information-gathering activities within the Ministry with a view to identifying the needs for modifying the core and complementary information systems for workforce planning and management. Based on the outcome of this study, the Ministry developed its computerized MIS in an integrated framework.

The Ministry was experiencing inordinate delays in the recruitment processes. At the instance of the policy-makers, the planning team assessed the status of the Ministry's workforce recruitment system, and identified steps towards improving its effectiveness and efficiency [28]. The study analysed the roles of various agencies in recruitment, and recommended how to optimize the functioning of each agency's internal functioning and how to strengthen the interaction among them. Based on the outcome of the study, the Ministry took actions to streamline the recruitment system and minimize the recruitment delay to the irreducible minimum level.

Yet another concern faced by the policy-makers was how to bring about organizational changes such as decentralization. In response to the policy-makers' vision of hospital autonomy at the regional level, the planning team organized a two-tier national workshop [29]. This highly effective two-tier workshop ushered in a significant shift in MoH hospital administration policy. A policy document was prepared through interactive sessions at multiple levels [5].

In the light of this workshop and subsequent follow-up meetings, the Ministry later formulated and issued a series of guidelines on hospital autonomy and introduced organizational and managerial reforms, which eventually led to a re-engineering of hospital management in Oman [30]. These guidelines paved the way for effective decentralization of health administration to the hospital level. This new policy helped inter alia to improve workforce management at the hospital level [6].

### Problems still unresolved

Due to political exigency to speed up Omanization of the physician category, the medical colleges have tried hard to increase their intake capacities. Unfortunately, the intake could not be increased as much as desired due to inadequacies in the number(s) of occupied beds relative to the number of students. Similar problems have been encountered with regard to nursing education, as well. This constraint is expected to be eased somewhat after the current five-year plan is implemented, as a few more hospitals are expected to be built during this period.

Some deficiencies do still exist in workforce management and planning systems, and these need to be overcome in order to further improve the effectiveness and efficiency of the health workforce [31]. The workforce plans prepared by the Ministry of Health have not so far been able to address adequately the workforce issues facing the entire nation, due to inadequate interaction with other health care providers. The Ministry has now established a national human resources for health observatory (ONHO) with the support of the WHO Regional Office for the Eastern Mediterranean [32]. The observatory is expected to help focus on the entire country's health workforce issues, generate broader understanding of these issues, undertake multicentric research investigations and help prepare national health workforce policies and plans. The workforce planning team has observed a few instances of skills mismatch, dissonance between education and service, performance management issues, retention problems and recruitment difficulties in certain categories due to international market factors. All these issues are now being addressed by the policy-makers and workforce planners [33].

## Discussion and evaluation

This case study has demonstrated the experience of an Eastern Mediterranean country that turned to workforce planning in a conducive political environment. The Sultanate's development plans, prepared in line with the Royal directives, have helped to form the public policies and the sociopolitical environment for all-round development of the state. The policy-makers and planners of the Ministry of Health have helped to shape the Ministry's health workforce development initiatives. In the background of such an encouraging environment, the planning team's extensive and intensive workforce planning efforts and the educators' diligent and persistent educational endeavours, as depicted in this case study, ensured adequacy of a suitable workforce in health institutions. This contributed to Oman's notable achievement in the health care system and its increased self-reliance in health workforce.

## Conclusion

The health workforce planning team's efforts in the Sultanate of Oman was able to help improve the health workforce system because of several factors. The workforce plans meshed well with the health plans. The planners had the full support of the top management; they made sincere efforts to collaborate with all relevant departments or institutions. The methodology used by the planners was objective and transparent, i.e. clearly elucidated and open for discussion and further development. The information used for planning was fairly accurate and reliable, thanks to a well-functioning health information system [34]. All assumptions made were clearly stated and well understood by policy-makers. The plan documents or the accepted recommendations were disseminated to all concerned with implementation. The planners received feedback about the actions taken on the plans. Mid-course corrections on the plans were made when unforeseen changes occurred or any of the assumptions were violated. An important lesson of the Omani experience is that the policy makers and programme managers should never treat workforce planning documents as mere paperwork, but use these as management tools to achieve further progress in all facets of health workforce development.

## Competing interests

The author declares that he has no competing interests.

## Authors' contributions

The author was responsible for initiating the workforce planning approach and implementing it with the close collaboration of top-level policy-makers, health planners, health administrators and educators and the support of the planning team. He prepared the case study, and hence is solely responsible for the facts and observations made in this article.
